# Hydroxy-Selenomethionine, an Organic Selenium Source, Increases Selenoprotein Expression and Positively Modulates the Inflammatory Response of LPS-Stimulated Macrophages

**DOI:** 10.3390/antiox11101876

**Published:** 2022-09-22

**Authors:** Joan Campo-Sabariz, Adriana García-Vara, David Moral-Anter, Mickael Briens, Mohammed A. Hachemi, Eric Pinloche, Ruth Ferrer, Raquel Martín-Venegas

**Affiliations:** 1 Departament de Bioquímica i Fisiologia, Facultat de Farmàcia i Ciències de l’Alimentació, Universitat de Barcelona (UB), Institut de Recerca en Nutrició i Seguretat Alimentària (INSA-UB), 08028 Barcelona, Spain; 2 Adisseo France S.A.S., 92160 Antony, France

**Keywords:** cytokine production, glutathione peroxidase, immune response, macrophage polarization, oxidative stress, phagocytosis, selenium deprivation, selenoprotein P, 2-hydroxy-(4-methylseleno)butanoic acid

## Abstract

The role of 2-hydroxy-(4-methylseleno)butanoic acid (OH-SeMet), a form of organic selenium (Se), in selenoprotein synthesis and inflammatory response of THP1-derived macrophages stimulated with lipopolysaccharide (LPS) has been investigated. Glutathione peroxidase (GPX) activity, GPX1 gene expression, selenoprotein P (SELENOP) protein and gene expression, and reactive oxygen species (ROS) production were studied in Se-deprived conditions (6 and 24 h). Then, macrophages were supplemented with OH-SeMet for 72 h and GPX1 and SELENOP gene expression were determined. The protective effect of OH-SeMet against oxidative stress was studied in H_2_O_2_-stimulated macrophages, as well as the effect on GPX1 gene expression, oxidative stress, cytokine production (TNFα, IL-1β and IL-10), and phagocytic and killing capacities after LPS stimulation. Se deprivation induced a reduction in GPX activity, GPX1 gene expression, and SELENOP protein and gene expression at 24 h. OH-SeMet upregulated GPX1 and SELENOP gene expression and decreased ROS production after H_2_O_2_ treatment. In LPS-stimulated macrophages, OH-SeMet upregulated GPX1 gene expression, enhanced phagocytic and killing capacities, and reduced ROS and cytokine production. Therefore, OH-SeMet supplementation supports selenoprotein expression and controls oxidative burst and cytokine production while enhancing phagocytic and killing capacities, modulating the inflammatory response, and avoiding the potentially toxic insult produced by highly activated macrophages.

## 1. Introduction

Macrophages are immune cells that have an important role in immune regulation. The activation of these cells by pathogens, exogenous molecules or tissue injury signals triggers an oxidative burst, resulting in an increase in reactive oxygen species (ROS), followed by the release of various mediators such as IL-1β, IL-6, and TNFα and arachidonic acid-derived prostaglandins, which initiate the inflammatory response [1,2]. However, the disruption of any of these inflammatory and oxidative mechanisms will lead to massive oxidation and a potentially toxic insult, leading to persistent inflammation [3].

Selenoproteins play an important role in reducing ROS generated during the oxidative burst, and therefore, in controlling and resolving the inflammatory process [3,4]. Although selenoproteins are generally classified as antioxidants, they exhibit a wide range of functions in inflammation and immunity (see [5] for a review). The selenoproteome consists of 25 selenoproteins, and selenium (Se) availability in the organism is essential for the expression of these proteins because they contain a selenocysteine residue in their active site. Carlson et al. [2] demonstrated that the glutathione peroxidase (GPX) family consists of the most abundant selenoproteins in mouse macrophages after determining the whole selenoproteome based on mRNA abundance. These authors also concluded that GPX1 was the most abundant isoform of GPX and that macrophages had relatively high levels of expression of the gene encoding selenoprotein P (SELENOP), described by Barrett et al. [6] as a selenoprotein with an important role in immune function. The importance of adequate levels of dietary Se that can be efficiently incorporated into selenoproteins has been demonstrated in cell culture models and animals experiment. It has been shown that limited Se leads to a decrease in the expression of many selenoproteins, an increase in the production of ROS, and proinflammatory cytokines and further chronic inflammation [6,7].

A growing body of research has suggested that the dietary form of Se is a major determinant of its efficiency for meeting the Se requirement in livestock [8]. In this context, organic forms of Se in poultry diets seem to have a range of important advantages over traditional sodium selenite, since organic Se can be stored in the animals’ tissues [8]. Recently, we reported that 2-hydroxy-(4-methylseleno)butanoic acid (OH-SeMet), an organic Se source widely used in poultry nutrition, is capable of supporting selenoprotein synthesis in Caco-2 cells, and that the increase in selenoprotein gene expression allows the cell to rapidly synthesize selenoproteins, resulting in enhanced protection against oxidative stress [9]. In the present study, the role of OH-SeMet as a source of Se in THP-1 derived macrophages was studied to explore the effect of this dietary source on immune cells. To study the effects of OH-SeMet on macrophages, we first established a model of Se deprivation by removing fetal bovine serum (FBS) from the cell culture medium because it naturally contains organic Se forms, mostly selenomethionine. Se-deprived cells were subsequently supplemented with OH-SeMet to study its capacity to support selenoprotein synthesis and to protect against oxidative stress. Furthermore, the capacity of OH-SeMet to modulate the immune response of macrophages stimulated with lipopolysaccharide (LPS) was investigated. Given that FBS could affect macrophage LPS-activation [10], experiments were performed both in the absence and presence of FBS.

## 2. Materials and Methods

### 2.1. Materials

Roswell Park Memorial Institute 1640 medium (RPMI), non-essential amino acids, sterile phosphate buffered saline (PBS), β-mercaptoethanol, LPS from *Escherichia coli* 0111:B4, N-acetyl-L-cysteine (NAC), and forbol-12-miristate-13-acetate (PMA) were supplied by Sigma (St. Louis, MO, USA). TRI-Reagent, penicillin, and streptomycin were supplied by Life Technologies (Carlsbad, CA, USA). FBS was purchased from GE Healthcare Life Sciences (Issaquah, WA, USA). Tryptic soy agar (TSA) was purchased from Thermo Fisher Scientific Oxoid (Hampshire, UK). OH-SeMet (Selisseo^®^) was provided by Adisseo France SAS (Antony, France). Tissue culture supplies were obtained from Costar (Cambridge, MA, USA).

### 2.2. THP-1 Cell Culture

The human monocytic leukemia cell line THP-1 provided by ATCC (Manassas, VA, USA) was cultured as previously described [11]. Cells were differentiated to macrophages with PMA at a concentration of 100 nmol/L in RPMI 1640 for 3 days.

### 2.3. Salmonella Enteritidis Culture

*Salmonella enterica* serovar Enteritidis (phage type 4; nalidixic acid-resistant strain; S. Ent) was provided by Ignacio Badiola from the Centre de Recerca en Sanitat Animal (CReSA, IRTA-UAB, Bellaterra, Spain) and prepared as previously described [12]. To prepare the inoculum, the bacteria were grown at 37 °C in TSA for 24 h and used in the exponential growth phase as determined by absorbance at 600 nm.

### 2.4. Se Deprivation and OH-SeMet Supplementation

**Figure 1:**
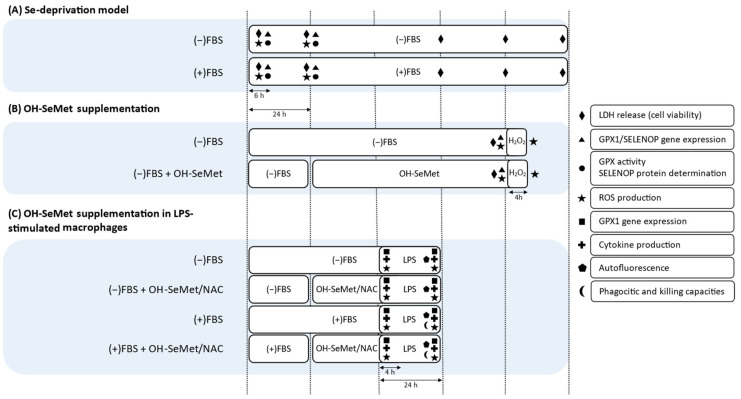
Experimental design for the Se-deprivation model, OH-SeMet supplementation, and LPS-stimulation model. (**A**,**B**) are explained in Section 2.4 and (**C**) in Section 2.5. FBS, fetal bovine serum; GPX, glutathione peroxidase; LDH, lactate dehydrogenase; LPS, lipopolysaccharide; NAC, N-acetyl-L-cysteine; OH-SeMet, 2-hydroxy-(4-methylseleno)butanoic acid; ROS, reactive oxygen species; SELENOP, selenoprotein P.

The model of Se deprivation was established on the basis that the only Se source in the culture medium was FBS [9]. To establish the Se-deprivation model, macrophages were maintained in the presence or absence of FBS for different periods of time (6 and 24 h, Figure 1A). Then, GPX activity, GPX1 gene expression, SELENOP protein and gene expression, as well as ROS production were determined as previously described [9].

GPX activity and SELENOP protein expression were determined from the cell supernatant after ultrasonic oscillation with a commercial kit GPX assay kit (Cayman Chemical, Ann Arbor, MI, USA) and a commercial ELISA kit (Cusabio, Wuhan, China) respectively, following the manufacturer’s instructions.

RT-PCR analysis was performed at the Centres Científics i Tecnològics of the Universitat de Barcelona (Barcelona, Spain) using the following primers: SELENOP (SELENOP_selenoprotein P1, Life Technologies) and GPX1 (GPX1_glutathione peroxidase 1, Life Technologies). After testing different reference genes, RPLP0 (RPLP0_ribosomal protein lateral stalk subunit P0, Life Technologies) was used for normalization purposes.

Intracellular ROS production was studied by the intracellular oxidation of 2′,7′-dichlorofluorescein to the fluorescent compound dichlorofluorescein and performed with a commercial intracellular ROS assay kit (OxiSelect, Cell Biolabs Inc., San Diego, CA, USA) following the manufacturer’s instructions.

Based on the results obtained in the absence of Se, deprivation for 24 h was chosen for OH-SeMet supplementation experiments (Figure 1B). Se-deprived macrophages were then supplemented with OH-SeMet at 12.5 μM and 625 μM for an additional 72 h. In the case of ROS production, cells were further incubated for 4 h with H_2_O_2_ 1 mM.

Cytotoxicity was evaluated by lactate dehydrogenase (LDH) assay performed as previously described [9]. Cell viability was not compromised in the absence or presence of FBS nor with OH-SeMet supplementation (data not shown). Given that there were differences in the number of cells in the cultures in the presence and absence of FBS, further results were normalized or expressed as mg protein.

### 2.5. OH-SeMet Supplementation in LPS-Stimulated Macrophages

After monocyte differentiation to macrophages, cells were maintained in the presence or absence of FBS for 24 h (Figure 1C) and then supplemented for 24 h with OH-SeMet at 12.5 μM and 625 μM or with NAC at 10 mM, a substrate with proven antioxidant activity [13]. These cells were then stimulated with two LPS concentrations (100 and 250 ng/mL) for 4 and 24 h. In these cells, GPX1 gene expression and ROS production were determined as in the former experimental model. To determine cytokine production, the concentrations of TNFα, IL-1β, and IL-10 in the culture medium were assayed with an enzyme-linked immunosorbent assay (ELISA) kit (Diaclone, Besançon, France), according to the manufacturer’s instructions.

The phagocytic and killing capacities of macrophages were investigated in cultures stimulated with 250 ng/m LPS for 24 h in the presence of FBS and supplemented with 625 µM OH-SeMet or 10 mM NAC. After the stimulation period, the cultures were inoculated with *Salmonella* Enteritidis at a multiplicity of infection (MOI) of 10 for 3 h. The phagocytic and killing capacities were evaluated based on bacterial disappearance from the incubation and intracellular medium, respectively. Colony-forming units (CFU) were counted in a sample of the incubation medium and in cell lysate, as previously described [12].

The autofluorescence emitted by macrophages at a wavelength of 561 nm was examined with a confocal laser scanning microscope (TCS-SP5; Leica Lasertechnik, GmbH, Germany) at the Centres Científics i Tecnològics of the Universitat de Barcelona. Images were processed using ImageJ software (public domain, version 1.41, National Institutes of Health).

### 2.6. Statistical Analysis

Results are given as means ± SEM. Significant differences were detected by one-factor ANOVA followed by Bonferroni’s post hoc test using IBM SPSS Statistics software, version 27.0 (SPSS Inc., Chicago, IL, USA). *p* < 0.05 was considered to denote significance.

## 3. Results

### 3.1. Se Deprivation and OH-SeMet Supplementation

**Figure 2:**
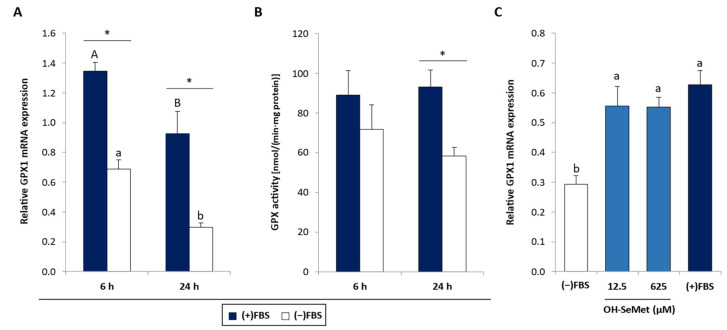
(**A**) GPX1 relative gene expression and (**B**) GPX activity in macrophages cultured in (+)FBS or (−)FBS for 6 or 24 h and (**C**) GPX1 relative gene expression in macrophages maintained for 24 h in (−)FBS or in (+)FBS and supplemented with 12.5 and 625 μM OH-SeMet for an additional 72 h. Gene expression results were calculated using 2^−ΔCt^. The results are expressed as mean ± SEM of n = 6 cultures. The asterisk (*) denotes significant differences (*p* < 0.05) between (+)FBS and (−)FBS (**A**,**B**), and different letters denote differences (*p* < 0.05) between incubation periods (**A**) and conditions (**C**).

In macrophages cultured in the absence of FBS, GPX1 gene expression was significantly reduced after 6 and 24 h with respect to macrophages cultured in the presence of FBS (Figure 2A). The data also revealed a decrease in this variable with incubation time. This reduction in GPX1 gene expression in the absence of FBS was accompanied by a reduction in GPX activity, but only after 24 h incubation (Figure 2B). Se-deprived cells supplemented with OH-SeMet (Figure 2C) showed a significant increase in GPX1 gene expression, reaching values similar to those obtained in the presence of FBS.

**Figure 3:**
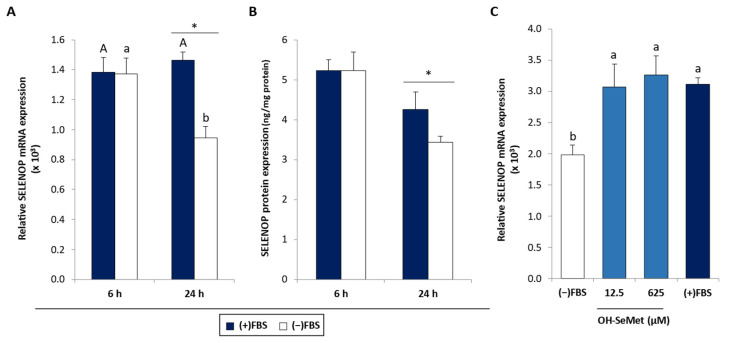
(**A**) SELENOP relative gene expression and (**B**) SELENOP protein expression in macrophages cultured in the presence (+) or absence (−) of FBS for 6 or 24 h and (**C**) SELENOP relative gene expression in macrophages maintained for 24 h in (+)FBS or in (−)FBS and supplemented with 12.5 and 625 μM OH-SeMet for an additional 72 h. Gene expression results were calculated using 2^−ΔCt^. The results are expressed as mean ± SEM of n = 6 cultures. The asterisk (*) denotes significant differences (*p* < 0.05) between (+)FBS and (−)FBS (**A** and **B**) and the letters denote differences (*p* < 0.05) between incubation periods (**A**) and conditions (**C**).

SELENOP gene expression was also reduced following FBS removal but only after 24 h incubation (Figure 3A), an effect that was accompanied by a reduction in SELENOP protein expression (Figure 3B). Here again (Figure 3C), OH-SeMet supplementation was able to return SELENOP gene expression to values similar to those obtained in the presence of FBS.

**Figure 4:**
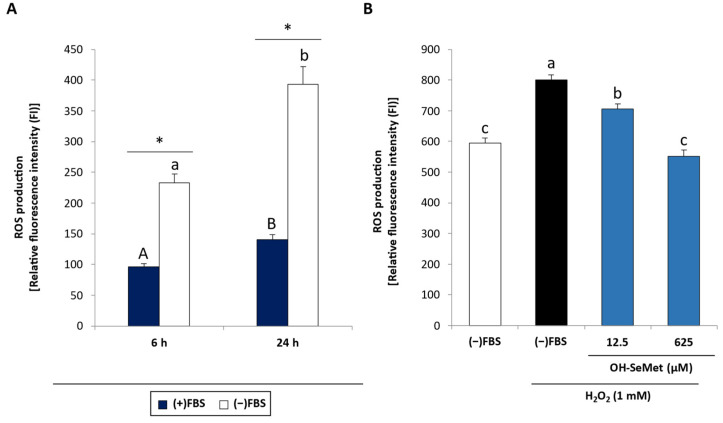
ROS production in (**A**) macrophages cultured in (+)FBS or (−)FBS for 6 or 24 h and (**B**) macrophages supplemented with 12.5 and 625 μM OH-SeMet for an additional 72 h and then stimulated with 1 mM H_2_O_2_ for 4 h. ROS production is shown as relative fluorescence intensity (FI) obtained at the beginning of the experiment ((FI_f_ × 100)/FI_i_). The results are expressed as mean ± SEM of n = 9 cultures. The asterisk (*) denotes significant differences (*p* < 0.05) between (+)FBS and (−)FBS (**A**) and different letters denote differences (*p* < 0.05) between incubation periods (**A**) and conditions (**B**).

FBS removal induced an increase in ROS production after 6 and 24 h incubation (Figure 4A). Moreover, ROS levels were significantly higher after 24 h when compared to 6 h incubation. When these cells were stimulated with 1 mM H_2_O_2_, 12.5 µM OH-SeMet conferred partial protection while 625 µM OH-SeMet was capable of completely counteracting the effect of H_2_O_2_, reaching values similar to non-stimulated cells (Figure 4B).

### 3.2. OH-SeMet Supplementation of LPS-Stimulated Macrophages

For the establishment of the LPS-stimulated macrophage model, the effect of LPS (100 and 250 ng/mL; 4 and 24 h stimulation) on GPX1 gene expression and ROS production in macrophages maintained in the absence of FBS was studied.

**Figure 5:**
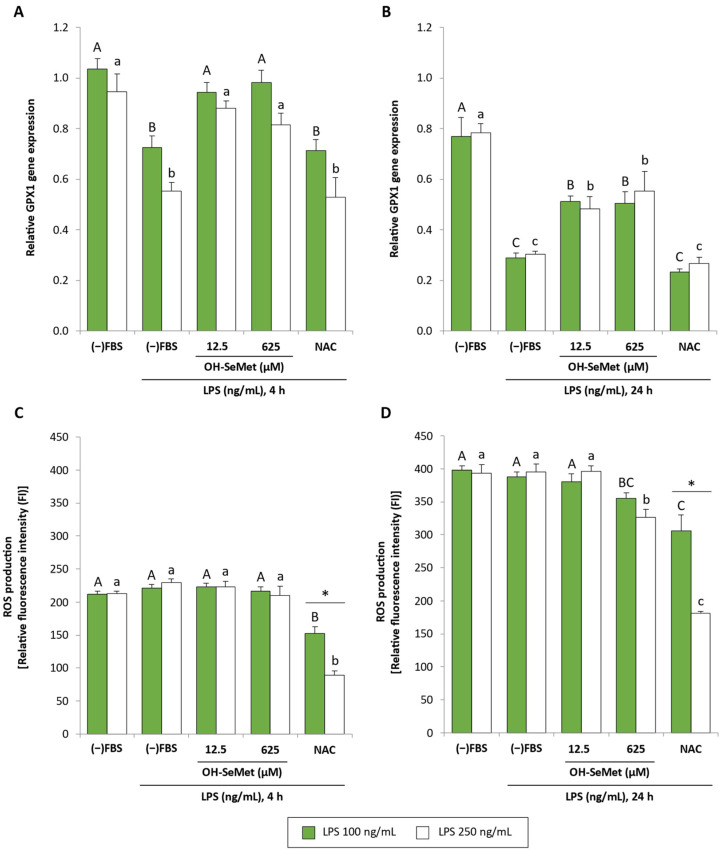
GPX1 relative gene expression (**A**,**B**) and ROS production (**C**,**D**) in macrophages maintained in (−)FBS for 24 h, supplemented for 24 h with 12.5 and 625 μM OH-SeMet or 10 mM NAC and then stimulated with LPS (100 and 250 ng/mL) for 4 or 24 h (**A**–**D**, respectively). Gene expression results were calculated using 2^−ΔCt^. ROS production results are shown as relative fluorescence intensity (FI) obtained at the beginning of the experiment ((FI_f_ × 100)/FI_i_). The results are expressed as mean ± SEM of n = 6–9 cultures. The asterisk (*) denotes significant differences (*p* < 0.05) between the two LPS concentrations used for each incubation period. Different letters denote significant differences (*p* < 0.05) between conditions for each LPS concentration.

GPX1 gene expression (Figure 5A,B) did not differ significantly between the two LPS concentrations tested. After 4 and 24 h simulation, a similar profile was observed: stimulation with LPS reduced GPX1 gene expression whereas supplementation with OH-SeMet completely or partially restored this parameter with respect to non-stimulated cells. In these conditions, NAC did not induce any effect. The results obtained regarding ROS production (Figure 5C,D) showed no effect after stimulation at any LPS concentration in either stimulation period. Interestingly, after 24 h stimulation, the data revealed that supplementation with 625 µM OH-SeMet reduced ROS production. NAC also reduced ROS production, this effect being more pronounced at the highest LPS concentration.

**Figure 6:**
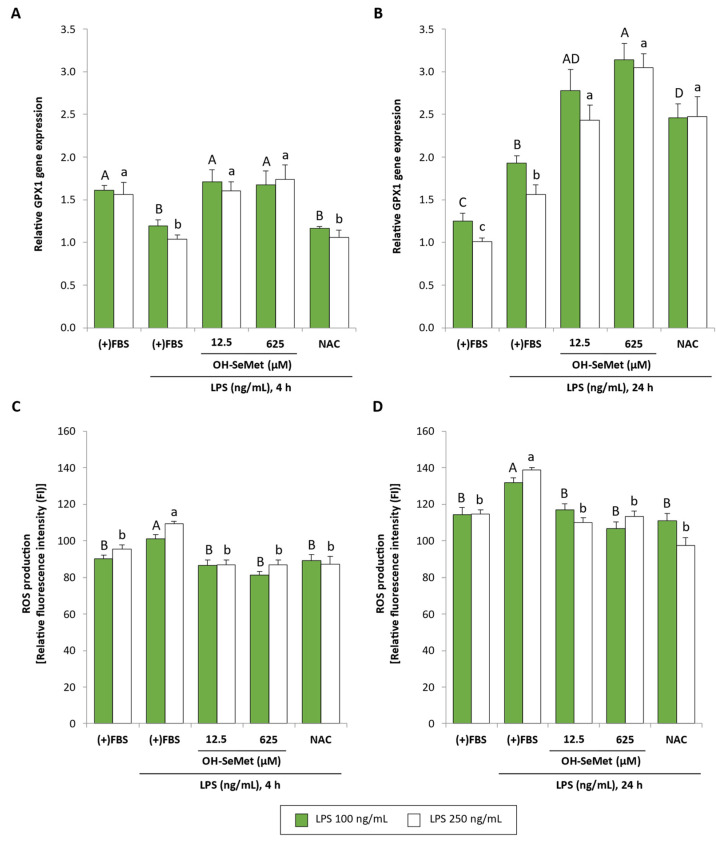
GPX1 relative gene expression and ROS production in macrophages maintained in (+)FBS for 24 h, supplemented for 24 h with 12.5 and 625 μM OH-SeMet or 10 mM NAC and then stimulated with LPS (100 and 250 ng/mL) for 4 or 24 h (**A**–**D**, respectively). Gene expression results were calculated using 2^−ΔCt^. ROS production is shown as relative fluorescence intensity (FI) obtained at the beginning of the experiment ((FI_f_ × 100)/FI_i_). The results are expressed as mean ± SEM of n = 6–9 cultures. The asterisk (*) denotes significant differences (*p* < 0.05) between the two LPS concentrations used for each incubation period. Different letters denote significant differences (*p* < 0.05) between conditions for each LPS concentration.

Given the lack of effect of LPS on ROS production, the effect of LPS stimulation on GPX1 gene expression and ROS production was also studied in the presence of FBS (Figure 6). In these conditions, no differences were observed between the two LPS concentrations used. After 4 h stimulation, a similar profile to cells maintained in the absence of FBS was observed (Figure 6A); here again, OH-SeMet completely restored GPX1 gene expression in contrast to NAC. However, after 24 h stimulation (Figure 6B), LPS increased GPX1 gene expression and OH-SeMet and NAC increased this parameter to values higher than those in non-stimulated cells. As for ROS production (Figure 6C,D), a similar profile was observed after 4 and 24 h stimulation: LPS increased ROS production whereas supplementation with OH-SeMet and NAC reduced this parameter to values similar to those in non-stimulated cells.

**Figure 7:**
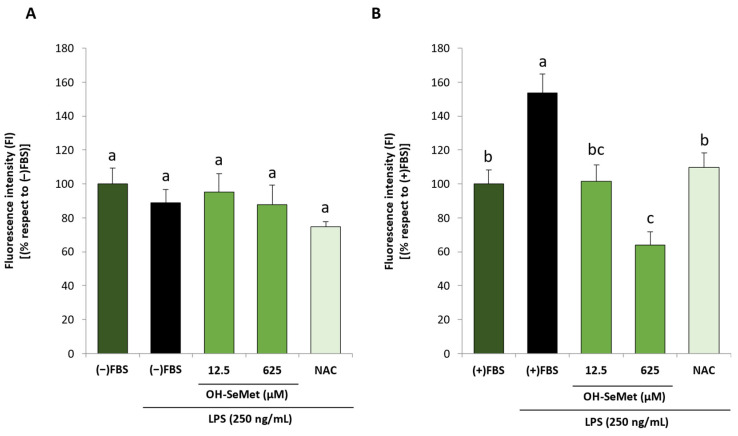
Autofluorescence in macrophages maintained in (−)FBS or (+)FBS for 24 h (**A**,**B**, respectively), supplemented for 24 h with 12.5 and 625 μM OH-SeMet or 10 mM NAC and then stimulated with LPS (250 ng/mL) for 24 h. The results are expressed as mean ± SEM of n = 12 macrophages. Different letters denote significant differences (*p* < 0.05) between conditions for each LPS concentration.

In further experiments, only the highest LPS concentration (250 ng/mL) was used. To better understand the lack of an LPS-induced effect on ROS production observed in the absence of FBS, the autofluorescence emitted by macrophages after LPS stimulation (24 h, 250 ng/mL) was studied as an oxidative stress indicator (Figure 7). In the absence of FBS, there were no significant changes in autofluorescence in any of the conditions tested (Figure 7A). However, in the presence of FBS (Figure 7B), incubation with LPS significantly increased autofluorescence intensity, and supplementation with OH-SeMet (both at 12.5 and 625 µM) decreased this parameter to values similar to those of non-stimulated cells or even lower, respectively. NAC also decreased autofluorescence to values similar to those of non-stimulated cells.

The effect of LPS on cytokine production was studied in macrophages cultured in the absence or presence of FBS. In non-stimulated cells (Table 1), TNFα, IL-1β, and IL-10 production in the absence and presence of FBS and after 4 and 24 h incubation was very low (below the detection limit) and, as expected, it increased after LPS stimulation. Cytokine production was significantly higher in the presence of FBS compared with the absence of FBS except for IL-1β at 24 h.

**Figure 8:**
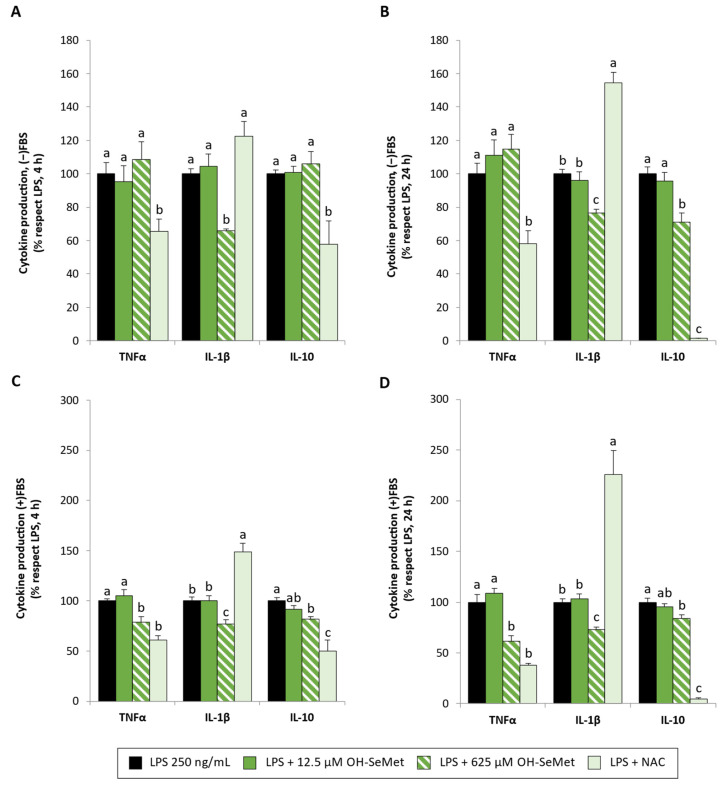
Cytokine production (TNFα, IL-1β, and IL-10) in macrophages maintained in (−)FBS or (+)FBS for 24 h (**A**–**D**, respectively), supplemented for 24 h with 12.5 and 625 μM OH-SeMet or 10 mM NAC and then stimulated with LPS (100 and 250 ng/mL) for 4 or 24 h (**A**–**D**, respectively). The results are expressed as mean ± SEM of n = 9 cultures. Different letters denote significant differences (*p* < 0.05) between conditions for each cytokine.

OH-SeMet supplementation (Figure 8) at 625 µM reduced IL-1β production in all conditions tested, as well as TNFα production but only in the presence of FBS in this case. However, NAC supplementation induced a reduction in TNFα production but a remarkable increase in the production of the proinflammatory cytokine IL-1β. OH-SeMet maintained IL-10 production at 4 h stimulation in the absence of FBS and slightly reduced its production in the other conditions (28.8% in the absence and 15.9–17.9% in the presence of FBS). In contrast, incubation with NAC induced a drastic decrease in IL-10 production (42.1–50.1% at 4 h and 95.7–98.6% at 24 h).

**Figure 9:**
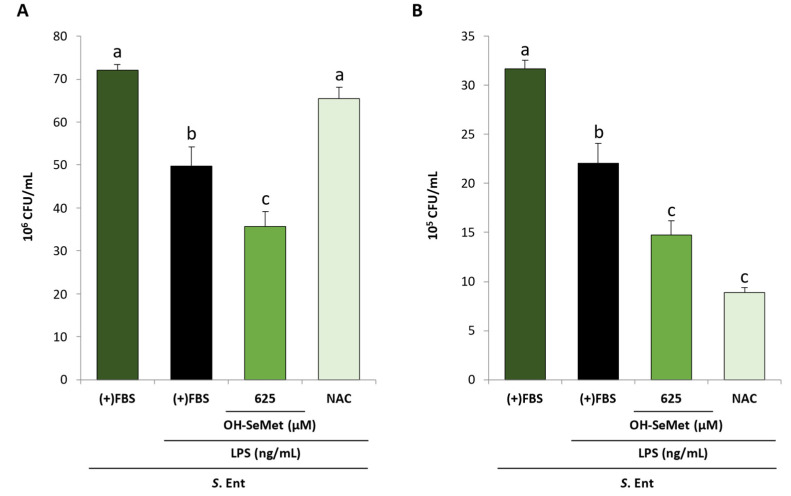
Extracellular (**A**) and intracellular (**B**) CFU counts in macrophages maintained in (+)FBS for 24 h, supplemented for 24 h with 625 μM OH-SeMet or 10 mM NAC, stimulated with LPS (250 ng/mL) for 24 h, and then infected with *Salmonella* Enteritidis (MOI 10) for 3 h. The results are expressed as mean ± SEM of n = 6 cultures. Different letters denote significant differences (*p* < 0.05) between conditions. CFU, colony-forming units; S. Ent, *Salmonella* Enteritidis.

Finally, the effect of OH-SeMet supplementation (625 µM) on the phagocytic and killing capacities of LPS-stimulated macrophages in the presence of FBS was studied after incubation with *Salmonella* Enteritidis (Figure 9). LPS stimulation reduced the number of CFU of *Salmonella* Enteritidis from samples of both extracellular and intracellular compartments. OH-SeMet supplementation accentuated this effect, suggesting an increase in the phagocytic and killing capacities. Meanwhile, NAC only decreased the CFU of *Salmonella* Enteritidis from the intracellular compartment, suggesting an increase in killing capacity.

## 4. Discussion

Se incorporation in animal diets is of great relevance, as Se supplementation is reported to be required for optimal growth and health [8]. In fact, inadequate dietary Se is considered a risk factor for several chronic diseases in animals and humans, associated with oxidative stress and immune response [5,14,15,16]. Recently, it has been demonstrated that a low Se status is related to a higher risk of death from COVID-19 [17,18]. Macrophage activation is regularly accompanied by an increase in the production of ROS and, for this reason, these cells require adequate levels of antioxidant defenses, such as selenoprotein expression and activity, in order to avoid their harmful effects when produced in excess. Therefore, Se reserves in the body are needed to maintain effective antioxidant defenses in stress conditions [8]. Moreover, Se levels in diet also affect the inflammatory signaling capacity and anti-pathogen activities of macrophages [7]. Prabhu et al. [19] and Vunta et al. [1,4] stated that GPX activity in macrophages is markedly decreased in Se deficiency. Accordingly, we found that the expression and/or activity of the two major selenoproteins in macrophages, firstly GPX1 and secondly SELENOP [2], were reduced in these conditions. Moreover, there is a correlation of these data with its gene expression, suggesting regulation at the transcriptional level. Nevertheless, translational regulation cannot be ruled out, since Schoenmakers et al. [20] stated that a Se-deficient environment decreases the expression of high-stress-related selenoproteins due to the reduction in the expression of a specific tRNA[Ser]Sec isoform (mcm5Um). GPX1 gene expression is a good biomarker of Se status, since it was markedly downregulated even at 6 h of Se deprivation, in parallel with the production of ROS. Indeed, it has previously been reported that Se deficiency increases ROS production [2,21], which could be related to this downregulation of selenoproteins. Given these results, the removal of FBS from the medium can be considered a useful model to simulate Se deficiency since it allows for the monitoring of selenoprotein behavior and thus oxidative stress modulation when Se levels are modified (deficiency and supplementation).

It has been proven that OH-SeMet is fully converted into selenomethionine, conferring the ability to increase Se deposition in tissues of all species, including in muscle of chickens [22,23,24], eggs and breast muscle of laying hens [25], muscle of growing pigs [26], sow milk [27], beef cattle [28,29], and dairy cows [30]. Thus, improving Se availability will help to improve health and to give rise to a biofortified animal products which are beneficial for human consumers. Therefore, although metabolism is complete in different tissues, it should be considered that conversion may be a limiting factor in a cell culture model. For this reason, relatively high non-toxic OH-SeMet concentrations have been used. In addition, pure organic forms of Se such as selenomethionine and OH-SeMet have a greater capacity to modulate selenoprotein gene expression than selenized yeasts or inorganic Se sources such as sodium selenite in different tissues [9,24,31,32,33]. In this context, it has previously been reported that OH-SeMet supplementation increased GPX1 and SELENOP gene expression in intestinal Caco-2 cells [9], consistent with the results obtained here in macrophages. It was also recently confirmed that Se supplementation and particularly OH-SeMet can support GPX activity when supplemented to poultry muscle tissues [34]. In the same way, Dhanjal et al. [3] found that GPX1 gene expression was upregulated in murine macrophages (RAW264.7) after incubation with an organic Se source extracted from wheat matrices, consistent with the results obtained here in macrophages and illustrating the capacity of this organic Se source to modulate gene expression in an immune cell. In the case of SELENOP, little information is available regarding the regulation of gene expression by Se in macrophages. However, it is well known that increased expression of SELENOP is induced when mouse macrophages switch from the M1 to M2 phenotype [35], thus highlighting the role of SELENOP in the resolution of inflammation, stated as well by Barret et al. [6], Short et al. [36], and Ding et al. [37].

Previous studies have demonstrated that organic Se sources confer a better protective effect than inorganic sources against different oxidative stress conditions [32,38,39,40,41]. Therefore, upregulation of GPX1 and SELENOP gene expression by OH-SeMet and a further decrease in ROS production induced by H_2_O_2_ could maintain effective antioxidant defenses and would help the cell to adapt to and overcome the stress with minimal negative consequences. It should be considered that an increase in ROS production triggers the formation of secondary oxidative products. Previously, we observed that OH-SeMet reduced protein carbonylation and lipid peroxidation in Caco-2 cells incubated with H_2_O_2_ [9].

The fact that OH-SeMet is able to increase the gene expression of these selenoproteins illustrates the capacity of this organic source to prepare the cell when the immune response starts. Therefore, we continued our study using an in vitro model of macrophages stimulated with LPS. In the first trial, we observed that FBS absence (Se-deficient model) affected macrophage response, because there were no changes in ROS production after LPS stimulation. Therefore, the effect of supplementation with OH-SeMet was also tested in the presence of FBS, which itself contains small amounts of selenomethionine [9]. It should be noted that the basal animal diet also contains low quantities of selenomethionine derived from cereals [42].

LPS significantly decreased GPX1 gene expression in virtually all conditions tested, as confirmed by Carlson et al. [2] and Wang et al. [43], who attributed this to dysregulation of selenogenome expression. Nevertheless, the pattern of GPX1 gene expression changed in the presence of FBS at 24 h, when higher expression of GPX1 was observed in the presence of LPS. Wang et al. [43] described that in LPS-stimulated RAW264.7 macrophages, an increase in some selenoproteins, such as TXNRD, was observed at 24 h, which may be a mechanism to counteract the effects of LPS during long incubation periods. The upregulation of GPX1 gene expression induced by OH-SeMet and the further reduction in ROS production shows the capacity of this organic source to support the expression of this selenoprotein even under non-physiological conditions. Regarding the effect of NAC, the decrease in ROS production was not accompanied by the upregulation of GPX1 gene expression in virtually any of the conditions tested, except for 24 h LPS stimulation in the presence of FBS. In this context, consistent with our results after a long period of incubation, Krifka et al. [44] reported that supplementation of NAC in RAW264.7 macrophages exposed to HEMA (a potent pro-oxidant) for long periods of time increased GPX1 and GPX2 expression. These authors attributed this upregulation to high levels of ROS accompanied by high levels of glutathione synthesized from NAC.

Regarding oxidative stress and cytokine production, these parameters varied when FBS was present or not in the culture: Firstly, in the presence of FBS, LPS induced the production of ROS, TNFα, IL-1β, and IL-10, as well as an increase in autofluorescence. Secondly, when FBS was removed from the medium, LPS had no effect either on ROS production or on autofluorescence, and there was a slight increase in cytokine production. Similarly, Palacio et al. [10] stated that in the absence of FBS, LPS was not capable of inducing ROS production in THP-1 macrophages. Nevertheless, consistent with our results, when these cells were incubated with LPS together with NAC, a decrease in ROS was observed. The lower production of cytokines observed was consistent with Safir et al. [45] who reported that the absence of FBS led to a loss of immune competence of J774.1 macrophages and/or the lower expression of proteolytic enzymes that are necessary for the release of cytokines such as TNFα from its precursor [45]. These results could also be attributed to the lack of an LPS-binding protein in the medium in the absence of FBS, which would be necessary for LPS to efficiently bind to its receptor (Toll-like receptor 4, TLR4) [46,47]. Nevertheless, although ROS production was not modified by the different conditions tested, the absence of FBS increased ROS production without increasing either autofluorescence or cytokine production, suggesting no association of this effect with macrophage activation. In contrast, when FBS is present, it has been reported that LPS efficiently activates the NF-κβ and MAPK pathways, producing changes in the redox state of the cell and increasing the secretion of several cytokines [11,48]. Moreover, Verstovsek et al. [49] observed an increase in autofluorescence in splenic macrophages stimulated with LPS as Sköld et al. [50] and Pankow et al. [51] observed in alveolar macrophages due to the presence of higher amounts of ROS as well as higher metabolic activity and Edelson et al. [52] due to the formation of protein adducts. Furthermore, autofluorescence is associated with macrophage activation [53] and with cytokine production, such as IL-1α [54].

Supplementation with OH-SeMet reduced ROS production, the autofluorescence peak, and cytokine production induced by LPS. Se-deficient cells usually show higher NF-κβ activation, with further higher expression of inflammatory cytokines [21,55]. In a similar way, it has been observed that Se reduced the production of proinflammatory cytokines by NF-κβ inhibition [56,57]. Regarding NAC, Ryan et al. [58] observed that this substrate was capable of both decreasing LPS-mediated IL-8 production as well as the translocation of NF-κβ to the nucleus in THP-1 derived macrophages. In contrast, we found an increase in IL-1β in all conditions when NAC was added to cells. Similar results were obtained by Parmentier et al. [13] who attributed this effect to NF-κβ activation. In the same way, Al-Shukaili et al. [59], also indicated that NAC upregulated the production of proinflammatory cytokines and downregulated anti-inflammatory cytokine production by peripheral blood mononuclear cells. All these results confirm the controversial effect of NAC, a substrate with proven antioxidant activity, in cytokine production [59]. Since IL-10 is described to inhibit IL-1β processing [60], the drastic loss of IL-10 produced by NAC found in our study could explain the failure to control IL-1β production in LPS-stimulated macrophages. Nelson et al. [61] stated that Se supplementation was able to induce the polarization of bone marrow-derived macrophages from M1 macrophages towards M2 macrophages. Several studies have shown that OH-SeMet supplementation in non-stressed conditions is capable of decreasing the production of proinflammatory cytokines such as IL-1β or TNFα and increase the expression of anti-inflammatory cytokines such as IL-10 [24,37,62,63,64]. Nevertheless, contrary to NAC, when a challenge was induced, OH-SeMet supplementation helped the macrophages to control the cytokine production by decreasing both proinflammatory cytokines (TNFα and IL-1β) and maintaining the anti-inflammatory cytokine IL-10, as previously described in other tissues [27,37,64]. It is important to remark that the control of IL-10 production is needed in stressed conditions since it is described that high IL-10 production leads to a suppression of the immune response and, therefore, a failure to control the infection [65].

Thus, OH-SeMet supplementation in non-stressed conditions would favor polarization to M2 macrophages. However, in stressed conditions, its protective effect would mainly consist of leading macrophages to be less prone to transition to the M1 phenotype. In fact, Mosser and Edwards [66] suggested another classification model in which macrophages have a functional plasticity that allows them to modify their function to assist in host defense, wound healing, and/or immune regulation, depending on the environmental signals. Thus, the OH-SeMet would optimize the macrophages activity to avoid excessive immune response or immune suppression that can cause damage or secondary infections, respectively. This could explain the improvement of phagocytosis and killing capacities by OH-SeMet compared to NAC. Xu et al. [21] stated that the phagocytic capacity decreased in macrophages that were deficient in Se, while Se supplementation increased these capacities [45,67,68]. In humans, it has been shown that different selenium sources activate the phagocytosis of tumor cells by macrophages (see [18] for a review). Moreover, it has been described that oxidative stress caused by Se deficiency could involve macrophage dysfunction [69]. Overall, the supplementation of these cells with OH-SeMet, which is capable of upregulating selenoprotein expression, would control oxidative damage and thus, phagocytosis. Since the phagocytic capacity of macrophages is among the key features of the innate immune response, these results highlight the capacity of OH-SeMet to foster an innate immune response with minimal negative consequences for the tissues. Such results corroborate the observations made in different in vivo trials [24,27,37,64], where OH-SeMet supplementation promoted innate immune response compared to sodium selenite and selenized yeast, and provide further knowledge on OH-SeMet’s mode of action on macrophages.

## 5. Conclusions

In conclusion, the supplementation of macrophages with OH-SeMet supported GPX1 and SELENOP gene expression and controlled the oxidative burst. Thus, OH-SeMet optimized and regulated the inflammatory response and thereby enhanced phagocytic and killing capacities of macrophages.

## Figures and Tables

**Table 1:** Cytokine production (TNFα, IL-1β and IL-10) in macrophages maintained in (−)FBS or (+)FBS for 24 h and then stimulated with LPS (100 and 250 ng/mL) for 4 or 24 h. The results are expressed as mean ± SEM of n = 9 cultures. The asterisk (*) denotes significant differences (*p* < 0.05) between (−)FBS and (+)FBS. ND, not detected.

		(−)LPS	(+)LPS	
Time	Cytokine (pg/µg)	(+/−)FBS	(−)FBS	(+)FBS
4 h	TNFα	ND	2.18 ± 0.159	15.21 ± 0.838 *
	IL-1β	ND	0.27 ± 0.021	0.43 ± 0.027 *
	IL-10	ND	0.02 ± 0.002	0.31 ± 0.023 *
24 h	TNFα	ND	7.02 ± 0.564	13.08 ± 0.742 *
	IL-1β	ND	0.99 ± 0.122	0.96 ± 0.039
	IL-10	ND	0.25 ± 0.014	1.07 ± 0.077 *

## Data Availability

Data are contained within the article.
